# A Scoping Review of Emergency Department Discharge Risk Stratification

**DOI:** 10.5811/westjem.2021.6.52969

**Published:** 2021-09-23

**Authors:** Todd A. Jaffe, Daniel Wang, Bosten Loveless, Debbie Lai, Michael Loesche, Benjamin White, Ali S. Raja, Shuhan He

**Affiliations:** * Massachusetts General Hospital and Brigham and Women’s Hospital, Harvard Affiliated Emergency Medicine Residency, Boston, Massachusetts; † Kansas City University School of Medicine, Kansas City, Missouri; ‡ Rocky Vista University College of Osteopathic Medicine, Ivins, Utah; § University College of London, Division of Psychology and Language Sciences, London, England; ¶ Massachusetts General Hospital, Department of Emergency Medicine, Boston, Massachusetts; || Harvard Medical School, Department of Emergency Medicine, Boston, Massachusetts

## Abstract

**Introduction:**

Although emergency department (ED) discharge presents patient-safety challenges and opportunities, the ways in which EDs address discharge risk in the general ED population remains disparate and largely uncharacterized. In this study our goal was to conduct a review of how EDs identify and target patients at increased risk at time of discharge.

**Methods:**

We conducted a literature search to explore how EDs assess patient risk upon discharge, including a review of PubMed and gray literature. After independently screening articles for inclusion, we recorded study characteristics including outcome measures, patient risk factors, and tool descriptions. Based on this review and discussion among collaborators, major themes were identified.

**Results:**

PubMed search yielded 384 potentially eligible articles. After title and abstract review, we screened 235 for potential inclusion. After full text and reference review, supplemented by Google Scholar and gray literature reviews, we included 30 articles for full review. Three major themes were elucidated: 1) Multiple studies include retrospective risk assessment, whereas the use of point-of-care risk assessment tools appears limited; 2) of the point-of-care tools that exist, inputs and outcome measures varied, and few were applicable to the general ED population; and 3) while many studies describe initiatives to improve the discharge process, few describe assessment of post-discharge resource needs.

**Conclusion:**

Numerous studies describe factors associated with an increased risk of readmission and adverse events after ED discharge, but few describe point-of-care tools used by physicians for the general ED population. Future work is needed to investigate standardized tools that assess ED discharge risk and patients’ needs upon ED discharge.

## INTRODUCTION

Emergency department (ED) discharge presents challenges and opportunities related to patient safety. Studies have demonstrated that gaps in ED discharge operations, including identification of high-risk patients and discharge processes, have led to multiple patient-safety concerns including poor comprehension of instructions, medication non-compliance, and lost to follow-up.^1^–^4^ Related work has investigated how interventions for specific disease states can improve the discharge process and follow-up.^5^ In other processes of care, clinical decision tools are now used frequently in the ED, specifically with regard to stratifying illness severity on presentation and risk-stratifying patients in need of additional laboratory studies or imaging.^6^–^9^

In the inpatient setting, appropriate discharge planning remains a core element of care coordination, and numerous studies have demonstrated the benefit of robust discharge planning processes, such as decreased readmission rates and increased prescription drug adherence.^10^–^13^ In the ED, however, the development of diligent processes of care surrounding discharge is limited by time and resource constraints. Recent studies have found ED-based discharge planning initiatives to improve patient comprehension, yet the link to improvement in clinical outcomes has been less defined.^14^ As the ED remains the safety net for many at-risk populations, it could be argued that appropriate discharge planning is of even greater concern. In fact, studies have found that only a fraction of patients being discharged from the ED reliably attend follow-up visits.^15^,^16^

How EDs identify patients at increased risk at time of discharge remains largely uncharacterized. There may be numerous methods by which EDs risk-stratify patients at time of discharge, yet a comprehensive review of the literature is limited. A scoping review of the literature may aid in identifying point-of-care discharge risk-stratification tools. We aimed to explore the medical literature to assess how EDs identify and target patients at increased risk at time of discharge. We further aimed to conduct a review of how ED clinicians may use point-of-care tools to discover patients at increased risk and the clinical factors that inform these risk-stratification tools.

## METHODS

We conducted a literature review and facilitated discussions with experts to determine how EDs assess patient risk upon discharge from the ED. Our prespecified search protocol was developed per collaboration among a medical research team consisting of attending and resident emergency physicians, research fellows, and medical students. We first searched the medical literature using PubMed for relevant articles published in the English language over the past 10 years, given the evolving landscape of emergency care. Initial inclusion criteria included articles that describe tools developed by emergency clinicians for discharge risk-stratification or those specifically related to patient discharge from the ED. We excluded articles that describe primary care, office-based, or inpatient initiatives as our focus remained on emergency care discharge planning and assessment. Additional exclusion criteria included any articles regarding pediatric discharge and studies greater than 10 years old. Initially, if there were any question of relevance to our research question, we erred on the side of inclusion.

**Figure 1:**
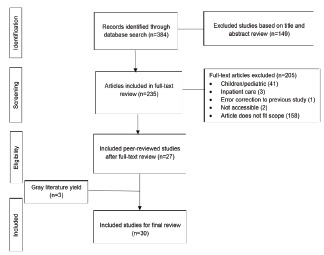
Flow diagram of review of discharge risk-stratification studies.

The initial PubMed search yielded 384 articles with potential for inclusion. Two authors (DW and BL) screened all titles and abstracts independently for potential relevance. Of these, we excluded 149 articles deemed out of scope based on preliminary title and abstract review, as well as duplicates and inaccessible articles. Of the articles that had potential for inclusion, the two authors conducted an independent, full-text review to determine eligibility and then met to resolve any disagreements. Of these articles, key data including title, author, description of study, and participants. A third author (TAJ) reviewed this consolidated list, and in collaboration with SH, DW and BL, filtered the articles according to whether they met our research aim. Figure 1 details the review process followed and outlines the exclusion criteria.

**Population Health Research Capsule**

What do we already know about this issue?

*Emergency department (ED) discharge presents challenges and opportunities related to patient safety, yet how ED clinicians perform discharge risk assessment is largely uncharacterized.*

What was the research question?

*How do ED clinicians identify and target patients at increased risk at the time of discharge?*

What was the major finding of the study?

*Many studies describe clinical risk factors but few describe point-of-care tools utilized by ED clinicians.*

How does this improve population health?

*By improving the understanding of ED discharge risk stratification tools, this study helps shed light on the lack of standardization of the process and potential harms that remain.*

We then conducted an additional full-text review and examined the references and related citations for all screened articles. We also searched Google Scholar and performed a Google search to identify high-quality, non-peer-reviewed (gray) literature related to our study. During full-text review, we extracted additional data from included articles including a comprehensive catalog of the patient and clinical factors included in discharge risk assessment. A more complete research protocol is included in the appendix. Based on this list, the primary author (TAJ) categorized the factors and reviewed categorization with additional members of the research team.

We conducted two additional meetings with emergency physicians in ED operations leadership roles to review findings and identify themes. These experts included one clinical operations director and an executive vice chair at a large academic ED, each of whom has numerous publications and national presentations on clinical operations, patient-centered communication, and discharge process improvement. Based on the literature review and expert meetings, major themes were cultivated and discussed for this review.

## RESULTS

The PubMed search yielded 384 potentially eligible articles for title and abstract review. These were initially screened, with pertinent study data recorded for 235 articles for potential inclusion. After title and abstract review, 27 articles were included for full-text and reference review. Based on the review of references, and Google Scholar and gray literature review, we included three additional articles in our review. A summary of the reviewed studies is presented in the Supplemental Table.^17^–^45^ The subset of studies that in particular describe tools for ED discharge risk-stratification are included in Table 1. We identified key themes from this review, which are described below. Clinical and patient factors associated with discharge risk were also extracted and included.

Theme 1: *Over 60% of the included studies describe post-discharge risk assessment, whereas the use of point-of-care risk assessment tools appears limited.*

Our review found numerous examples of post-discharge risk assessment in the literature. Frequently, studies examined the safety of discharge via cohort studies of discharged patients from the ED and assessment of their post-discharge course. These studies were often specific to one disease-state or patient population. A few of these studies are highlighted below.

Gabayan et al performed a case-control study to assess the factors associated with poor outcomes in the elderly discharged from the ED. They found that multiple factors present at discharge including systolic blood pressure less than 120 millimeters mercury at discharge, heart rate greater than 90 beats per minute, and poorer score on a mini mental status exam all increased likelihood of intensive care unit (ICU) admission for patients greater than age 65.^21^ Noel et al performed a cohort study to delineate which clinical factors and patient characteristics were associated with increased seven-day mortality after ED discharge.^41^ They found that older age, male gender, and evidence of pre-existing conditions were all associated with increased risk of seven-day mortality among discharged ED patients.

More commonly, studies assessed adverse events after discharge for one specific patient population or condition. For example, Chang et al examined how psychiatric illness may relate to early death after ED discharge.^47^ The authors found that the presence of a psychiatric diagnosis in patients discharged from the ED was independently associated with a greater likelihood of death compared with those without a psychiatric diagnosis. Atzema et al performed a study of patients with atrial fibrillation discharged from the ED and the factors associated with 90-day death for these patients.^48^ The authors concluded that lack of follow-up care had a correlation with increased risk of death at 90 days.

Theme 2: *Of the point-of-care tools that exist, inputs and outcome measures varied, and few were applicable to the general ED population.*

As our primary aim was to identify what risk-assessment tools are used by emergency clinicians at the time of discharge, we conducted a thorough review to identify studies describing the use of these tools. Few were elucidated in our primary literature review, yet our gray literature review highlighted the limited tools that have been used by emergency clinicians. Table 2 describes the risk assessment tools that were identified and the studies related to their use. Three of the risk-stratification tools are further described below.

Gabayan et al conducted a retrospective cohort study leading to the development of a risk score to help predict short-term outcomes of patients following ED discharge.^20^ The authors examined patient and clinical factors associated with two clinical outcomes: inpatient admission and ICU admission/death. They then used these factors to develop a score to help predict each of the outcomes. Inputs were related to age, body mass index, systolic blood pressure, heart rate, comorbidities, length of stay, and evidence of recent inpatient admission. The authors retrospectively reviewed patient data to inform the development of the score; however, the ongoing use of the tool has not yet been described.

Meldon et al examined the use of a five-question screening tool to predict return ED visits, admission, or nursing home placement among discharged patients from the ED.^43^ The authors found that patients deemed high risk based on this screening tool were more likely to have the composite outcome of repeat ED presentation, admission, or nursing home placement. The study specifically focused on the elderly population and included demographic and clinical questions.

Schrader et al published an observational study in 2019 describing the inputs to and development of a tool to identify patients at risk of “discharge failure.” The authors defined discharge failure related to return ED visits and/or lack of adherence to primary care provider or specialist follow-up.^44^ The authors incorporated this information, as well as data from previous studies regarding other patient and clinical factors related to adverse events after ED discharge, to inform the development of their tool. These factors included patient gender, insurance status, Emergency Severity Index score, and vitals on discharge. The authors conducted simultaneous observational studies to train and test their tool and found that the tool may have application for predicting discharge failures. The ongoing use of the tool has yet to be studied.

The aim of these risk-assessment tools varied. As indicated in Table 2, most were designed to assess risk of readmission, risk of poor compliance with follow-up, and/or risk of adverse events. Some tools incorporated the measurement of risk of ED re-presentation, whereas others assessed risk of readmission to the hospital and/or the ICU. One study also measured increased discharge risk as evidenced by frequent ED visits (>3) over the course of six months after ED discharge. Furthermore, the inputs included in the risk scores or clinical tools varied. Multiple studies targeted the elderly population, with whom the use of functional assessment questions was common. Vital signs were also commonly used as a proxy for discharge risk assessment, in addition to the occasional use of gender, insurance status, and whether or not the patient had a recent admission, among other inputs. Few, if any, of these studies described the risk-assessment tools as a means to assess needs at discharge, specifically what resources are needed and the urgency of follow-up.

Secondary analysis aimed to characterize which factors were commonly used among reviewed risk assessment tools. Of the reviewed articles 60% (18/30) included risk assessment modalities, including those at point-of-care and post-discharge. After data retrieval of the factors included in the reviewed papers, we categorized the findings into the six most common groupings: vital signs; change in mental status; age; recent ED visit or admission; medical complexity; and social complexity (Table 2). Medical complexity includes references to specific diagnoses, the presence of chronic disease (most commonly pulmonary, cardiac, or renal impairment), higher acuity triage, and polypharmacy. Social complexity refers to housing instability, limited assistance at home, or Medicaid as insurance. Of these articles 83% (15/18) highlighted increased age as a risk factor, with 3/4 point-of-care tools including increased age as an input. Fifty percent (9/18) included medical complexity as a marker of increased discharge risk assessment, with vital signs (22%), change in mental status (22%), social complexity (28%), and recent ED visit or admission (28%) being less commonly referenced. Rarely, gender and physician or nursing concern were included as an input into increased discharge risk.

Theme 3*: Many studies describe initiatives to improve the discharge process, including improving comprehension and medication review. However, there remains a paucity of literature describing assessment of post-discharge resource needs.*

Numerous studies highlighted the importance of thorough discharge planning, including initiatives to improve patient comprehension. Other studies emphasized the importance of multidisciplinary involvement in the discharge process, whereas additional studies documented the importance of early discharge planning to decrease ED length of stay.

Moss et al described the use of a multidisciplinary team to aid in the discharge planning process and care coordination of patients, with the goal of improving their post-acute care return to the community.^31^ The authors found that patients who underwent the intervention had a decreased rate of readmission to the hospital, and ED staff had a high rate of satisfaction with the program. Other studies explored the use of tools to facilitate close follow-up, often targeting specific patient populations that had historically been deemed higher risk. Biese et al conducted a study to assess the impact of a telephone follow-up for elderly patients by trained nurses after discharge from the ED.^46^ They found no significant improvement with the intervention as evidenced by a lack of change in readmission rate or adverse events when compared with the control group, yet responses to the study highlighted the limitation that the telephone follow-up may not have been appropriately intensive or did not target the correct population.

Studies regarding initiatives aimed at improving the discharge process frequently described targeting a specific disease process or at-risk population. Although eight studies included in our review described initiatives to improve discharge comprehension, there was a notable lack of standardized tools to identify the patient’s post-discharge needs.

## DISCUSSION

In our review of the use of discharge risk-assessment tools in the ED, we found multiple applications described in the literature. Many studies detailed post-discharge risk assessment tools for specific patient populations and medical conditions seen in the ED. Few studies described risk-assessment tools that could be used at the point of care, and the tools that do exist were often limited to the elderly population. Only one study in our review described a point-of-care risk-assessment tool for the broad ED population, and the use of that clinical tool remains in its nascency. Of the studies that included discharge risk assessment, a variety of patient and clinical factors were commonly referenced. In particular, studies frequently highlighted increased age as a risk factor, as well as medical complexity including chronic cardiac or renal impairment and taking multiple medications. Vital sign abnormalities, changes in mental status, social complexity, and recent ED visits or admission were also referenced, although less frequently.

Previous studies have been limited to examining discharge risk assessments for specific patient populations or focusing on inpatient care. Lowthian et al conducted a systematic review of the discharge of elderly patients from the ED and found no significant benefit associated with the development of ED-based community transition strategies for the geriatric population.^49^ Moons et al conducted an analysis of four discharge risk-assessment tools specifically for the elderly population, and found one tool to be more accurate than others.^50^ Schwab et al expanded on this study and conducted a systematic review of the available elderly discharge risk-assessment tools and found two of these – Identification of Seniors at Risk and Triage Risk Stratification Tool – to be the best validated. Yet, these studies solely examined the elderly population and are not applicable to the broader ED population.

Our study expands on this work by providing a thorough review of ED discharge risk assessment tools for the broader ED population. Furthermore, our study provides multiple takeaways regarding ED discharge which may merit further exploration. First, we found that a wide array of studies document patient risk factors when being discharged from the ED. These studies define and examine risk with a variety of endpoints including risk of readmission, adverse events, and mortality. This augments the previous literature as we have documented the lack of standardization among these assessments, both in how they define discharge risk and the patient populations they examine. Most notably, we found that there remains a paucity of available point-of-care risk- assessment tools that are designed for the general ED population. Furthermore, how these tools are used by emergency clinicians, and how they can predict the resources needed for patients post-discharge, remains unknown.

From our study, we were able to elicit some commonalities among the risk-assessment tools referenced in the literature. Specifically, we found that the elderly population is commonly included in risk-assessment modalities as well as targets for intervention from ED discharge. Other factors were also commonly cited including medical complexity and, less commonly, social concerns and other clinical factors. The tools that exist, however, often included a variety of these inputs and rarely targeted a general ED population. Furthermore, the outcomes they studied often varied. These outcomes included repeat ED visits, hospital admission, patient comprehension, and 30- and 90-day mortality.

We also considered which clinical factors, at a minimum, should be included in a discharge risk-assessment tool. Our study found that age was commonly associated with increased discharge risk, as well as the presence of complex medical illness, vital sign abnormalities, altered mental status, recent admission, and social concerns. It is likely that at the very least these inputs are foundational for an accurate discharge risk-assessment tool; however, more research and discussion are likely needed to inform the development of a standardized tool. With the significant variability in both the factors included in discharge risk assessment as well as the measured outcomes, perhaps a first aim would be to develop consistency among the potential varying definitions of discharge risk. This may enable more standardization related to the testing of the clinical and patient factors included in the tools referenced above. Our study sheds light on the potential outcome measures and factors that may be included in these studies and provides a review of the disparate literature that currently exists.

The importance of safe ED discharge warrants further exploration. Challenges and improvements with the ED discharge process have been associated with harm and better patient outcomes, respectively.^1^,^2^,^15^ Better understanding of the tools that exist to assess ED discharge risk helps shed light on the lack of standardization of the process and potential harms that remain. Our review suggests that the methodology for ED discharge risk assessment varies immensely, evidenced by the wide array of clinical factors used in the risk-assessment tools described.

Although many studies documented risk factors for readmission and adverse events, few studies detailed the tools that may be used at the point of care, and even of these that exist, outcome measures varied. Of these point-of-care tools, there were some commonalities that likely contribute to their potential use at the point of care. Two of the point-of-care tools included screening questions used to predict discharge failure, whereas the other two tools included scores calculated from inputs of patient and clinical factors. Obtaining data that encompass these scores likely requires additional time at the point of care, such as reviewing mode of transportation, insurance status, and recent ED presentations. Validation related to the novel tools is also relatively limited as validation studies were only conducted at the study sites. Further research is indicated to externally validate the novel risk scores to further assess both their accuracy and application.

It is also unclear from our study whether a single point-of-care tool would be able to capture both risk of readmission or adverse events as well as discharge needs. It may be that tools that identify patients at increased risk of readmission or adverse events may inform clinicians of those patients requiring more intensive discharge needs assessment. Additionally, as we have seen success with discharge process improvements targeting specific disease states, it may be challenging to develop a tool that applies to a broader patient population. Future studies may help to identify standardized tools for ED discharge risk assessment of all patients, as well as investigate which tools may help assess patient needs upon ED discharge.

## LIMITATIONS

Our study is not without limitations. First, although our aim was to identify how EDs identify and target patients at increased risk upon discharge, we found only four point-of-care tools in our review. Furthermore, two of the point-of-care tools have been published in the last three years and thus have no long-term validation. Although our review was conducted using both published and gray literature, there may be other discharge risk-assessment tools being used by ED clinicians that have not yet been described. We attempted to mitigate this by discussion with clinicians with significant experience in ED operations; however, this limitation remains.

The use of expert reviewers also presents limitations. Although they hold leadership positions in ED operations and have published on the topic, our expert reviewers may have limitations in their knowledge on the topic. Furthermore, their input may also introduce unintentional bias from expert opinion. Our use of a standardized data collection tool aimed to include only objective data for our reviewed studies, yet that limitation remains. Given our goal was to scope the literature related to ED discharge risk assessment, we purposely aimed to capture heterogeneous studies. As a result, the use of a meta-analysis was not appropriate for our study. We addressed this by keeping a broad scope and by including rigorous methods to capture pertinent studies.

## CONCLUSION

In this clinical review of medical literature regarding ED discharge risk assessment, we found numerous studies describing patient risk factors associated with increased risk of readmission and adverse events after discharge from the ED, but few studies that describe point-of-care tools used by ED clinicians. Future work is needed to investigate standardized tools that assess ED discharge risk and patients’ needs upon ED discharge. Prospective studies on the use of these tools are needed to evaluate impact on patient outcomes.

## Supplementary Information





## Figures and Tables

**Table 1:** Select point-of-care emergency department discharge risk assessment tools.

Study title	Author	Population	Description of tool	Outcome measure	Inputs
A risk score to predict short-term outcomes following emergency department discharge	Gabayan, G	All ED patients	Developed a score based on coefficient estimates of the model variables	General inpatient admission, ICU admission/Death within 7 days	Age, BMI, SBP, HR, CCI, ED LOS, inpatient admission in the previous week.
A brief risk-stratification tool to predict repeat emergency department visits and hospitalizations in older patients discharged from the ED	Meldon, S	Elderly	Five-question screening tool for elderly patients (Triage Risk Stratification Tool)	Composite endpoint of subsequent ED use, hospital admission, or nursing home admission at 30 and 120 days.	Cognitive impairment, Difficulty walking, >4 meds, ED use in previous 30 days, or hospitalization in previous 90, RN professional recommendation.
Identifying diverse concepts of discharge failure patients at emergency department in the US: a large-scale, retrospective observational study	Schrader, C	All ED patients	Shout Score: Observational study to inform the development of a tool to assess and predict discharge failure	Return ED visits and/or lack of adherence to PCP or specialist follow-up	Gender, race, PCP assigned (y/n), homelessness, insurance status, means of arrival, vital signs, ESI, history of chronic conditions (y/n).
Return to the emergency department among elders: patterns and predictors.	McCusker, J	Elderly	Identification of Seniors at Risk (ISAR)	Return ED visit within 30 days and frequent ED visits, which include three or more within six months	Functional status, hospitalization within 6 months, visual impairment, mental impairment, multiple medications.

*ED*, emergency department; *BMI*, body mass index; *SBP*, systolic blood pressure; *HR*, heart rate; *CCI*, chronic condition indicator; *LOS*, length of stay; *RN*, registered nurse; *PCP*, primary care provider; *ESI*, Emergency Severity Index.

**Table 2:** Factors commonly included in discharge risk assessment.

Paper title	First author	Vital signs	Change in mental status	Age	Recent ED visit or admission	Medical complexity	Social complexity
Are vital sign abnormalities associated with poor outcomes after emergency department discharge?	Chang CY	x					
Qualitative factors in patients who die shortly after emergency department discharge	Gabayan G		x	x			
A risk score to predict short-term outcomes following emergency department discharge	Gabayan G	x		x	X		
Poor outcomes after emergency department discharge of the elderly: a case-control study	Gabayan G	x	x	x			
Effectiveness of a post-emergency department discharge multidisciplinary bundle in reducing acute hospital admissions for the elderly	Ong CEC			x			
Emergency department discharge diagnosis and adverse health outcomes in older adults	Hastings SN					x	
Tele-follow-up of older adult patients from the Geriatric Emergency Department Innovation (GEDI) program	Morse L			x			
Unscheduled return visits with and without admission post emergency department discharge	Hu KW			x		x	
Value of information of a clinical prediction rule: informing the efficient use of healthcare and health research resources	Singh S			x		X	
Unplanned early return to the emergency department by older patients: the Safe Elderly Emergency Department Discharge (SEED) project	Lowthian J			x			
A multidisciplinary care coordination team improves emergency department discharge planning practice	Moss JE			x	X	x	x
Short-term outcomes of elderly patients discharged from an emergency department	Denman SJ			x			
Factors associated with short-term bounce-back admissions after emergency department discharge	Gabayan GZ			x		x	x
Patterns and predictors of short-term death after emergency department discharge	Gabayan GZ			x		x	
Predictors of admission after emergency department discharge in older adults	Gabayan GZ			x		x	
A brief risk-stratification tool to predict repeat emergency department visits and hospitalizations in older patients discharged from the emergency department	Meldon, S		x	x	X	x	x
Identifying diverse concepts of discharge failure patients at emergency department in the USA: a large-scale retrospective observational study	Schrader, C	x			X	x	x
Return to the emergency department among elders: patterns and predictors.	McCusker, J		x	x	X	x	x
Total number of papers that include risk assessment factor		4	4	15	5	9	5
