# Supplementary material for: A Scoping Review of Emergency Department Discharge Risk Stratification
Source: West J Emerg Med. 2021 Sep 23;22(6):1218–26. doi: 10.5811/westjem.2021.6.52969 (PMC8597698; doi:10.5811/westjem.2021.6.52969)
Supplement: Supplementary file 1 [file wjem-22-1218-s001.docx]

**Supplemental Table.** Summary of reviewed articles.

| **Study Title** | **First Author** | **Journal (abbreviation)** | **Publication Year** | **Number of Participants** | **Type of Study** | **Description of Discharge Risk Assessment** |
| --- | --- | --- | --- | --- | --- | --- |
| Are vital sign abnormalities associated with poor outcomes after emergency department discharge? | Chang CY | Acute Med | 2019 | N/A | Case-Control | Any vital sign abnormality at ED discharge associated with over three-fold increase in likelihood of death within 15 days of ED discharge |
| Unmet needs at the time of emergency department discharge | Samuels-Kalow M | Acad Emerg Med | 2016 | 51 | Cross-sectional | Participants across multiple literacy groups identified actionable areas for improvement in the ED discharge process. These included the use of simplified/lay language, increased visual learning, and complete information. |
| Qualitative factors in patients who die shortly after emergency department discharge | Gabayan G | Acad Emerg Med | 2013 | 290,092 | Cross-sectional | Many factors associated with mortality including change in mental status, recent fall in the elderly, ill-appearing, malfunctioning indwelling device, presenting symptoms remain. |
| A risk score to predict short-term outcomes following emergency department discharge | Gabayan G | West J Emerg Med | 2018 | 104,025 | Retrospective Cohort | Developed a score based on coefficient estimates of the model that included age, BMI, SBP, HR, CCI, ED LOS, inpatient admission in the previous week. |
| Poor outcomes after emergency department discharge of the elderly: a case-control study | Gabayan G | Ann Emerg Med | 2016 | 600 | Case Control | Used mini mental exam, six-item screener, BP<120, pulse rate greater than 90 beats/min to assess risk after discharge. |
| Effectiveness of a post-emergency department discharge multidisciplinary bundle in reducing acute hospital admissions for the elderly | Ong CEC | Eur J Emerg Med | 2019 | 647 | Retrospective Cohort | The SAFE program was effective in reducing first acute hospital admissions in selected elderly and functionally challenged patients after ED discharge at 30 and 60 days. |
| Emergency department discharge diagnosis and adverse health outcomes in older adults | Hastings SN | J Am Geriatr Soc | 2009 | 1,851 | Retrospective Cohort | ED discharge diagnosis may improve methods of identifying individuals at high risk for problems after ED discharge and may inform the development of targeted interventions to reduce adverse health outcomes in vulnerable populations. |
| Tele-follow-up of older adult patients from the Geriatric Emergency Department Innovation (GEDI) Program | Morse L | Geriatrics (Basel) | 2019 | 57 | Retrospective Cohort | Both short-term and longer term follow-up telephone calls promoted the identification of needs by geriatric nurse that arise at different stages post-discharge. |
| Improving front-end flow in an urban academic medical center emergency department: the emergency department discharge facilitator team | Sharma R | J Urban Health | 2013 | 4,472 | Retrospective Cohort | A discharge facilitator team consisting of an ED attending, PA, and RN were able to identify/treat a group of patients who could be rapidly managed and discharged; associated with a reduction in length of stay for all low-acuity patients despite an increasing patient volume. |
| Unscheduled return visits with and without admission post emergency department discharge | Hu KW | J Emerg Med | 2012 | 413 | Retrospective Cohort | Old age, high-grade triage, and doctor-based factors were found to be significant predictors for unscheduled return visits, whereas advanced staff experience and ED crowding were not. |
| Value of information of a clinical prediction rule: informing the efficient use of healthcare and health research resources | Singh S | Int J Technol Assess Health Care | 2008 | n/a | Cross-sectional | A clinical prediction rule to assess patients who may be eligible for early discharge with chest discomfort; outcome measure was inappropriate ED discharge. |
| Capturing emergency department discharge quality with the Care Transitions Measure: a pilot study | Sabbatini AK | Acad Emerg Med | 2019 | 410 | Cross-sectional | Utilization of the Care Transitions Measure-3 (CTM-3) to assess transition of care from ED discharge. Investigators assessed how scores on this tool related to multiple outcome measures. |
| Examining patient comprehension of emergency department discharge instructions: Who says they understand when they do not? | Lin MJ | Intern Emerg Med | 2015 | 75 | Cross-sectional | Found that patients’ perceptions of their understanding of discharge instructions may not correlate with actual comprehension, specifically, college-educated patients and men. |
| Unplanned early return to the emergency department by older patients: the Safe Elderly Emergency Department Discharge (SEED) project | Lowthian J | Age Ageing | 2016 | 959 | Prospective Cohort | Older patients had higher rate of re-presentation with mixed reports regarding living alone, functional dependence, and polypharmacy. |
| A multidisciplinary Care Coordination Team improves emergency department discharge planning practice | Moss JE | Med J Aust | 2002 | 2,532 | Prospective Cohort | Targeted frail elderly patients, those living alone, homeless, complex medical issues, or frequent ED visits for care coordination efforts. |
| Short-term outcomes of elderly patients discharged from an emergency department | Denman SJ | J Am Geriatr Soc | 1989 | 200 | Prospective Cohort | Elderly patients had worse outcomes, were more likely to follow-up, and had worse functional impairments after discharge. |
| Older patients' understanding of emergency department discharge information and its relationship with adverse outcomes | Hastings SN | J Patient Saf | 2011 | 92 | Randomized Control Trial | Elderly patients had difficulty understanding discharge instructions with increase in adverse events for those who did not comprehend instructions. |
| Patient understanding of emergency department discharge instructions | Logan PD | South Med J | 1996 | 159 | Randomized Control Trial | Patients endorsed difficulty understanding home care and return instructions at ED discharge. |
| The effect of a clinical decision support for pending laboratory results at emergency department discharge | Driver BE | J Emerg Med | 2019 | 67,287 | Retrospective Cohort | Pending laboratory tests at time of discharge were pervasive, even when providers were prompted to review all pending studies prior to discharge. |
| Should let them go? Study on the emergency department discharge of patients who attempted suicide | Shin H | Psychiatry Investig | 2018 | 504 | Retrospective Cohort | Many patients who attempted suicide were discharged against medical advice in Korea; case management services may be beneficial to target this population. |
| Common stepwise interventions improved primary care clinic visits and reduced ED discharge failures: a large-scale retrospective observational study | Schrader CD | BMC Health Serv Res | 2019 | 227,627 | Retrospective Cohort | Providing insurance and designating primary care providers at time of discharge may improve follow-up and reduce ED discharge failures. |
| The provision of diagnosis at emergency department discharge: a pilot study | Wen LS | Emerg Med J | 2013 | 797 | Retrospective Cohort | Assessed whether patients are discharged with a pathological diagnosis and poses whether pathological diagnosis may affect clinical outcomes or patient satisfaction. |
| Factors associated with short-term bounce-back admissions after emergency department discharge | Gabayan GZ | Ann Emerg Med | 2013 | 5,035,833 | Retrospective Cohort | Factors associated with bounce-backs include elderly, Medicaid patients, and end stage renal disease. |
| Emergency department discharge reports as a management tool of clinical information | Conesa A | Med Clin (Barc) | 2003 | 11,188 | Retrospective Cohort | Found that ED discharge reports often had written information that was analyzed to be difficult to interpret. |
| Patterns and predictors of short-term death after emergency department discharge | Gabayan GZ | Ann Emerg Med | 2011 | 475,829 | Retrospective Cohort | Found high-risk discharge diagnoses including non-infectious lung disease, renal disease, and ischemic heart disease. Also including increased age, male gender, and comorbidities. |
| Pharmacist-led transition of care pilot targeting older people after emergency department discharge | Pearson SM | Sr Care Pharm | 2020 | 170 | Retrospective Cohort | Used a pharmacist-led transitional care, telephonic intervention pilot targeting older people to see whether that impacted composite of repeat ED visits, hospitalizations, or deaths within 30 or 90 days of discharge. |
| Predictors of admission after emergency department discharge in older adults | Gabayan GZ | J Am Geriatr Soc | 2015 | 505,315 | Retrospective Cohort | Assessed ED discharge factors associated with readmission, which included older age and history of renal and cardiac impairment. |
| A brief risk-stratification tool to predict repeat emergency department visits and hospitalizations in older patients discharged from the emergency department | Meldon, S | Acad Emerg Med | 2003 | 650 | Retrospective Cohort | Five-question screening tool for elderly patients (Triage Risk Stratification Tool), assessed TRST ability to predict the composite endpoint of subsequent ED use, hospital admission, or nursing home admission at 30 and 120 days. |
| Identifying diverse concepts of discharge failure patients at emergency department in the US: a large-scale, retrospective observational study | Schrader, C | BMJ Open | 2019 | 227,627 | Retrospective Cohort | Shout Score: Observational study to inform the development of a tool to assess and predict discharge failure. |
| Return to the emergency department among elders: patterns and predictors. | McCusker, J | Acad Emerg Med | 2000 | 1,122 | Retrospective Cohort | Identification of Seniors at Risk score: Assessed return ED visit within 30 days and frequent ED visits which include three or more within six months. |

*ED*, emergency department; *BMI*, body mass index; *SBP*, systolic blood pressure; *HR*, heart rate; *CCI*, chronic condition indicator; *LOS*, length of stay; *PA*, physician assistant; *RN*, registered nurse.
