# Supplementary material for: A Scoping Review of Emergency Department Discharge Risk Stratification
Source: West J Emerg Med. 2021 Sep 23;22(6):1218–26. doi: 10.5811/westjem.2021.6.52969 (PMC8597698; doi:10.5811/westjem.2021.6.52969)
Supplement: Supplementary file 2 [file wjem-22-1218-s002.docx]

Appendix**.** Search strategy for scoping review of discharge risk stratification.

Criteria for Inclusion: Articles that describe tools developed by emergency clinicians for discharge risk stratification or those specifically related to patient discharge from the ED.

Exclusion: We excluded those articles that described primary care, office-based or inpatient initiatives as our focus remained on emergency care discharge planning and assessment. Additional exclusion criteria included any articles that involved pediatric discharge and studies greater than 10 years old.

**Search Conducted, Pubmed 8/26/20:**

(((("emergency service, hospital"[MeSH Terms] OR (("emergency"[All Fields] AND "service"[All Fields]) AND "hospital"[All Fields])) OR "hospital emergency service"[All Fields]) OR ("emergency"[All Fields] AND "department"[All Fields])) OR "emergency department"[All Fields]) AND (((((("discharges"[All Fields] OR "discharging"[All Fields]) OR "patient discharge"[MeSH Terms]) OR ("patient"[All Fields] AND "discharge"[All Fields])) OR "patient discharge"[All Fields]) OR "discharge"[All Fields]) OR "discharged"[All Fields]) AND ("risk"[MeSH Terms] OR "risk"[All Fields]) AND "tool"[All Fields]

Time: 8/26/11 to 8/26/20

Yield: 384 articles

**Search Conducted, Gray Literature: 8/28/20**

**Google Search:** ("emergency"[All Fields] OR "emergency department"[All Fields]) AND OR ("patient"[All Fields] AND "discharge"[All Fields])) OR "discharge"[All Fields]) AND ("risk"[MeSH Terms] OR "risk"[All Fields]) AND "tool"[All Fields]

- Reviewed first 50 results
- Three additional studies/articles identified, which had not yet been included
- Categorized in similar matter as above
